# RSDB: A rare skin disease database to link drugs with potential drug targets for rare skin diseases

**DOI:** 10.1038/s41597-022-01654-2

**Published:** 2022-08-26

**Authors:** Tien-Chueh Kuo, Pei-Hua Wang, Yu-Ke Wang, Chia-I. Chang, Ching-Yao Chang, Yufeng Jane Tseng

**Affiliations:** 1 The Metabolomics Core Laboratory, Center of Genomic Medicine, National Taiwan University, No. 1, Sec. 4, Roosevelt Road, Taipei, 10617 Taiwan; 2 Graduate Institute of Biomedical Electronics and Bioinformatics, College of Electrical Engineering and Computer Science, National Taiwan University, No. 1, Sec. 4, Roosevelt Road, Taipei, 10617 Taiwan; 3 Department of Computer Science and Information Engineering, College of Electrical Engineering and Computer Science, National Taiwan University, No. 1, Sec. 4, Roosevelt Road, Taipei, 10617 Taiwan

**Keywords:** Bioinformatics, Drug development, Target identification, Databases

## Abstract

Rare skin diseases include more than 800 diseases affecting more than 6.8 million patients worldwide. However, only 100 drugs have been developed for treating rare skin diseases in the past 38 years. To investigate potential treatments through drug repurposing for rare skin diseases, it is necessary to have a well-organized database to link all known disease causes, mechanisms, and related information to accelerate the process. Drug repurposing provides less expensive and faster potential options to develop treatments for known diseases. In this work, we designed and constructed a rare skin disease database (RSDB) as a disease-centered information depository to facilitate repurposing drug candidates for rare skin diseases. We collected and integrated associated genes, chemicals, and phenotypes into a network connected by pairwise relationships between different components for rare skin diseases. The RSDB covers 891 rare skin diseases defined by the Orphanet and GARD databases. The organized network for each rare skin disease comprises associated genes, phenotypes, and chemicals with the corresponding connections. The RSDB is available at https://rsdb.cmdm.tw.

## Abstract (summary)

**Table:** 

Measurement(s)	Relationships between chemicals and genes • Relationships between diseases and genes • Relationships between diseases and phenotypes • Relationships between genes and phenotypes
Technology Type(s)	The Comparative Toxicogenomics Database (CTD) and DrugBank • DisGeNET, UniProt, The Comparative Toxicogenomics Database (CTD), Orphanet, ClinGen, Genomics England, NCBI ClinVar, The Human Phenotype Ontology (HPO), the GWAS Catalog, GWASdb28, the LHGDN and BeFree system • The Human Phenotype Ontology (HPO) and Genetic and Rare Diseases Information Center (GARD)
Sample Characteristic - Organism	Homo sapiens

## Background & Summary

Rare diseases affect fewer than 1 in 200,000 people in the U.S. or 1 in 2,000 people in Europe^1,2^. Although most rare diseases are complex, disabling, and life-threatening^3^, they lack related studies and approved treatments^4^ due to the limited prevalence and market^5^. Skin diseases cause significant nonfatal disability worldwide^6^, especially in resource-poor regions^7^. However, far little attention has been given to rare skin diseases^8^. In addition to the physiological burden, skin diseases’ economic and social impacts significantly lower patients’ quality of life^9,10^. Therefore, this work aims to help link drugs to drug targets for rare skin diseases.

Two databases, Orphanet and GARD, provide curated information on the diagnosis and currently available treatments for rare diseases^11^. Orphanet (www.orpha.net) covers rare diseases and orphan drugs, gathering and providing complete information and knowledge to improve diagnosis^12^. GARD, the Genetic and Rare Diseases Information Center, is a National Center for Advancing Translational Sciences (NCATS) program in the United States. It was established by the National Institutes of Health (N.I.H.) to provide information about symptoms, prevalence statistics, causes, treatments, diagnosis, and the latest research resources for over 6500 rare diseases^13^.

Although genetics accounts for the various causes of skin symptoms, the challenges of rare skin diseases are that they cannot be easily classified as skin disorders with a fixed set of symptoms. These symptoms vary from disease to disease and among patients with the same disease. Epidermolysis bullosa (E.B.) is a family of devastating rare skin diseases with friction inflicting painful, open wounds within the skin and internal epithelial tissue blistering^14–16^. Recent E.B. research has led to identifying mutations in 10 different genes^17,18^. One of the most severe forms of E.B. is recessive dystrophic epidermolysis bullosa (RDEB), caused by mutations in a protein called collagen VII^19^. Collagen VII provides the skin with structural integrity. There are over 500,000 people worldwide who suffer from this debilitating disorder. Simply looking at a single mutation or open wound would not help identify the disease or the treatment. Expansion of the symptoms (phenotypes) to look for more probable treatment is needed.

Drug repurposing can reduce the risk of failure and the massive cost of money and time in drug development by identifying new indications for an existing drug that is already approved^20,21^. Drug repurposing aims to find new relationships between the drug and disease^22^. However, related data regarding rare skin diseases are scattered and stored in several biomedical databases. Most patient-centered databases provide diagnostic criteria or currently available treatments and prognoses. We collected and integrated associated genes, chemicals, and phenotypes into a network to find novel drug-disease relationships for rare skin diseases. The rare skin disease database (RSDB) covers 891 rare skin diseases defined by the Orphanet and GARD databases. The organized network for each rare skin disease comprises associated genes, phenotypes, and chemicals connected via associations found in PubChem^23^, MeSH^24^, the Comparative Toxicogenomics Database (CTD)^25^, and Human Phenotype Ontology (HPO)^26^. The RSDB is available at https://rsdb.cmdm.tw.

## Methods

We collected data from public databases containing curated, inferred, literature-based information to create a database for connecting biomedical information. With curated disease genes, phenotypes, and phenotype genes as the direct molecular signatures of rare skin diseases, this work tries to link potential drugs to candidate rare skin disease targets with matched genes through disease-gene or disease-phenotype-gene relationships.

Currently, the RSDB contains 891 rare skin diseases, 28,077 genes, 9,732 phenotypes and 17,297 compounds with 16,411 disease-gene relationships, 15,793 disease-phenotype relationships, 12,184 disease-reference relationships, 641,789 gene-phenotype relationships, 17,636 gene-reference relationships and 61,282 references. The RSDB will be updated twice a year in June and December.

**Fig. 1:**
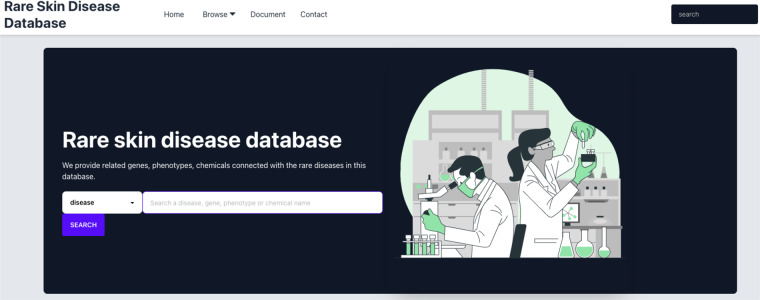
Search engine and homepage of the RSDB.

Users can visit the RSDB homepage (https://rsdb.cmdm.tw) to explore the data for rare skin disease information. On the RSDB website, users can access records and perform searches (see Fig. 1).

### Chemicals

A total of 17,297 environmental chemicals including approved drugs, were imported from the dataset of chemicals to genes in the CTD and DrugBank. All chemicals associated with genes are included in the RSDB.

### Diseases

Rare skin diseases were collected from Orphanet and GARD. Orphanet provides the disease classifications. All the rare diseases classified to the skin class were parsed and stored in the database.

The skin disease category was derived from NIH GARD. To determine whether a disease is a rare skin disease, we compared Orphanet as a basis. All information was downloaded, including the synonyms, definitions, inheritance, prevalence, and genes related to the disease. According to the NIH GARD, we found that 619 skin diseases have been described.

### Genes and disease-gene relationships

Associated disease-gene relationships were collected from DisGeNET v7^27^. DisGeNET provides three tiers: (1) expert-curated information, (2) inferred information, and (3) text-mining information. Expertly curated information was collected from UniProt, the CTD, Orphanet, ClinGen, and Genomics England. (2) Inferred information was collected from NCBI ClinVar, HPO, the GWAS Catalog, and GWASdb^28^. (3) Text-mining information was collected from the LHGDN and BeFree system.

### Phenotypes and disease-phenotype and gene-phenotype relationships

Associated phenotypes were collected from HPO and GARD. HPO provides disease-phenotype and gene-phenotype information. GARD provides rare disease-to-phenotype relationship information. We downloaded the 2020-12 version.

### References

Associated references were collected from the literature section of PubChem, which is linked to PubMed.

### Source database

All data from different public databases were collected as follows.

### Expertly curated information

UniProt^29^, the CTD^30^, Orphanet^31^, ClinGen^32^, Genomics England^33^

The CTD includes manually curated data on how chemicals interact with genes and proteins. Specifically, a chemical compound may interact with a gene or protein and influence its expression, folding, localization, activity, binding, abundance, and metabolic processing.

### Inferred information

NCBI ClinVar^34^, HPO^26^, the Genome-Wide Association Study (GWAS) Catalog^35^.

### Literature-based information

The literature-derived human gene-disease network (LHGDN)^36^, BeFree system^37^.

## Data Records

All the data files in RSDB are stored in the Synapse repository (10.7303/syn34512708)^38^ and are available under the terms of CC BY-NC-SA 4.0 (https://creativecommons.org/licenses/by-nc-sa/4.0/).

There are 22 CSV files in the repository. Among them are nine files describing the basic components in RSDB, including compounds, genes, phenotypes, etc. The other 13 files store the pairwise relationships between components.

We designated an internal ID for all the files to every entry in the first column. For the files describing basic components, associated properties like names, descriptions, and ID numbers from other databases will be stored in the following columns. For the files describing relationships, we separate the many-to-many relationships in RSDB into multiple entries of pairwise relationships. For example, disease_gene_relationships.csv stores internal disease ID and gene ID in the first and second columns, respectively. Disease with internal ID 3 is linked to the genes with internal ID 3 and 4 in the third and fourth entries. One can refer to diseases.csv and genes.csv for more information about the disease and genes involved in the relationships.

## Technical Validation

The datasets were retrieved from several public databases. According to the source database, the information we provide is curated by an expert or inferred from the literature or experiments. For example, our database connected four genes to the rare skin disease “exfoliative ichthyosis”: CSTA, KRT1, KRT2, and SERPINB8. Mutation in CSTA, which encodes cystatin A, can cause the disease^39,40^. Genetic linkages between the disease and KRT1 and KRT2, encoding keratin 1 and 2^41^, respectively. Loss-of-function mutations in SERPINB8, encoding serpin B8, are also linked to exfoliative ichthyosis^42^. The rare skin disease “epidermolytic palmoplantar keratoderma” has been confirmed to be caused by mutations in KRT1^43^, KRT9^44^, and KRT16^45^. This literature, which proves the accuracy of the disease-gene relationships in our data, is also provided to users via links to PubMed.

Here we demonstrate how our database can help drug repurposing using the well-known case of diacerein. Diacerein is a symptomatic drug in osteoarthritis. Its active metabolite, Rhein, decreases inflammation, reduces damage, and promotes the formation of new cartilage^46^. Diacerein has been effective against epidermolysis bullosa (EB) in the past decade by reducing blister counts and increasing skin stability^47^. There are four main types of EB, namely EB simplex (EBS), junctional EB (JEB), dystrophic EB (DEB), and Kindler syndrome (KS), according to the current international consensus classification. In RSDB, five genes directly link to chemical diacerein: ACAN, COL1A1, COL2A1, and IL1B. Among them, COL1A1 and COL2A2 are linked to “dystrophic epidermolysis bullosa” (DEB) and “localized dystrophic epidermolysis bullosa, pretibial form,” a subtype of DEB, respectively. This validates our data and shows the possibility of finding a potential drug for repurposing.

The RSDB includes all the pairwise relationships between disease, gene, phenotype, and chemical-disease and chemical-gene associations. For a particular rare skin disease, the profile of the disease and lists of associated genes, phenotypes, or chemicals are provided along with network visualization. Integrated information that only multiple searches across several databases can obtain is organized into one webpage. Crosslinks to other databases and related articles in PubMed facilitate further analysis and study.

One outstanding feature of the RSDB is network visualization. Diseases, phenotypes, genes, and chemicals are denoted by pink squares, gray triangles, blue circles, and orange hexagons, respectively. For the network containing more than 50 nodes, the CiSE layout^48^ will be applied to generate several circular layouts for each type of node to visualize the entire network without overlapping nodes. Otherwise, the fCoSE layout^49^ will be applied. In addition, several layout algorithms, including circle, concentric, and CoSE layouts, are also available for users to change different network layouts. To access the node name and the link to the node page, users can click on the node, and the node information and link will appear in the tooltip. To pan, zoom in and out of the network, a navigation toolbar is provided on the top-left of the network. Network visualization helps users find genes and phenotypes relevant to particular rare skin diseases.

**Fig. 2:**
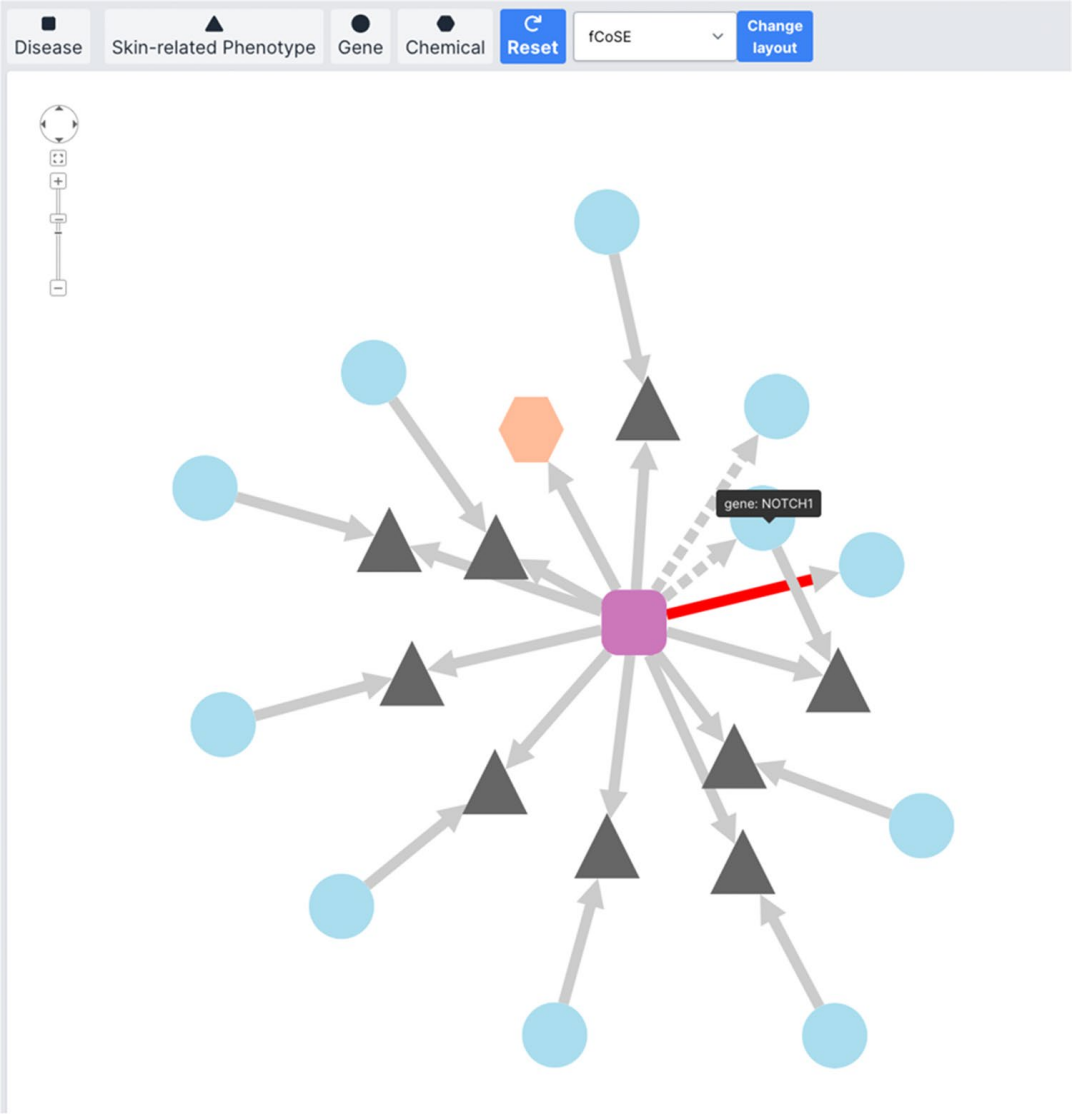
Screenshot of the network for cutis marmorata telangiectatica congenita (ORPHA: 1556). Nine phenotypes, two genes, and one chemical are directly connected to the disease, and eight genes are indirectly linked to the disease through phenotypes. The *NOTCH1* gene is related to the disease both directly and indirectly. Red edge stands for the curated disease-gene information.

A gene can be indirectly linked to a disease in the network if both nodes are connected to the same phenotype, an intermediate node. For example, the gene “NOTCH1”, shown in Fig. 2, links to the disease directly and indirectly through a phenotype with HPO ID 25107. Multiple sources that lead to the same connection between one pair of diseases and genes imply a strong relationship between the disease and gene. We hope these findings help scientists find promising research targets and accelerate orphan drug discovery.

We developed a disease-centered database covering 891 rare skin diseases with associated genes, phenotypes, and chemicals. We deployed a complete text search engine to include exact matches and fuzzy searches for the search terms. On each chemical/disease/gene/phenotype page, all associated chemical/disease/gene/phenotype information is connected and visualized in the network. In the associated chemical/disease/gene/phenotype tables, all associated data will be listed with data source and evidence. The associated data can be filtered with keywords via the top-right search form of the tables.

## Usage Notes

### Overview of the RSDB

**Fig. 3:**
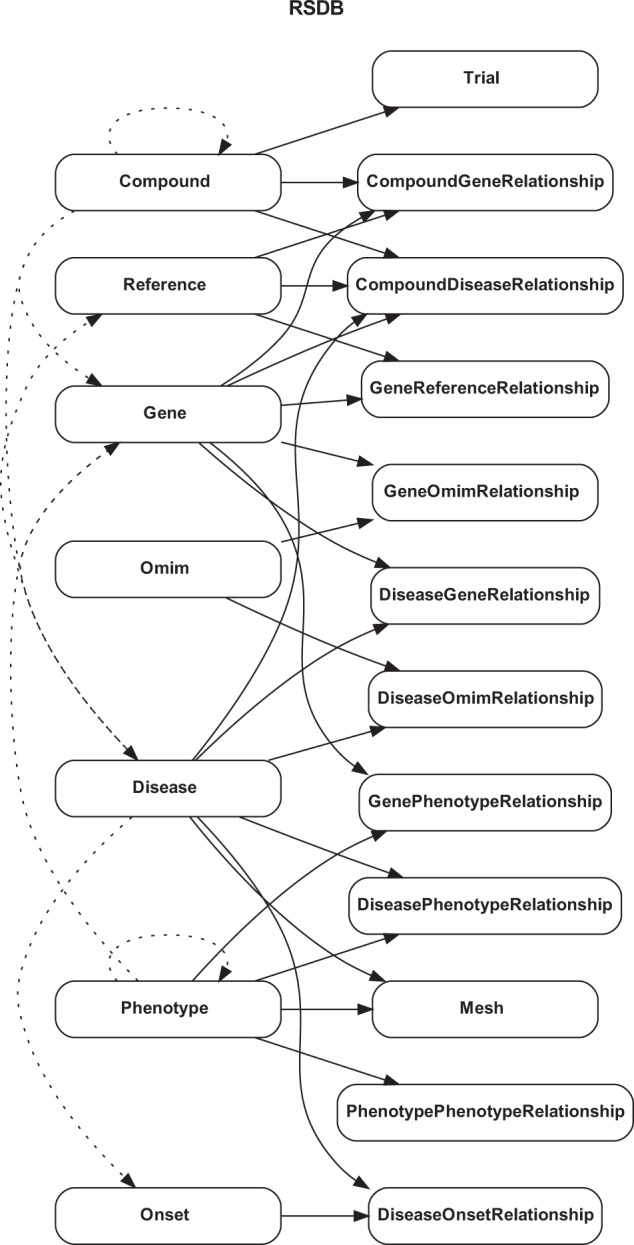
Entity-relationship diagram of the RSDB.

We designed the RSDB with critical components, including (1) rare skin diseases, (2) genes, (3) phenotypes, and (4) chemicals. All four elements were collected from manually curated databases and connected with the associated information. All related information of one disease is seen as the molecular signature of the disease. An entity-relationship diagram is displayed in Fig. 3.

## Data Availability

The code supporting this study’s findings is available on GitHub at https://github.com/CMDM-Lab/rsdb_publication. The scripts and packages used for the RSDB rely on open-source packages such as Ruby on Rails, MariaDB, ElasticSearch, Cytoscape.js^50^, and in-house Ruby scripts.
